# Beyond medication: Understanding child and caregiver perspectives on multifaceted adherence in pediatric hematopoietic stem cell transplantation

**DOI:** 10.1017/S1478951526102090

**Published:** 2026-03-23

**Authors:** Madeline Peek, Kathryn Vannatta, Misty Evans, Kimberly Taylor, Rajinder Bajwa, Ahna Pai, Cynthia A. Gerhardt, Micah A. Skeens

**Affiliations:** 1 The Abigail Wexner Research Institute, Nationwide Children’s Hospital, Columbus, OH, USA; 2 Department of Pediatrics, The Ohio State University College of Medicine, Columbus, OH, USA; 3 School of Nursing, Vanderbilt University, Nashville, TN, USA; 4 Sarah Cannon Cancer Center, Nashville, TN, USA; 5 Division of Hematology/Oncology/HCT, Nationwide Children’s Hospital, Columbus, OH, USA; 6 Center for Biobehavioral Health, Research Institute at Nationwide Children’s Hospital, Columbus, OH, USA; 7 Department of Pediatrics and Psychology, The Ohio State University, Columbus, OH, USA; 8 Center for Biobehavioral Health, Abigail Wexner Research Institute, Nationwide Children’s Hospital, Columbus, OH, USA

**Keywords:** Adherence, hematopoietic stem cell transplant, adherence barriers, medication adherence, adherence facilitators, pediatrics

## Abstract

**Objectives:**

This qualitative study sought to explore the unique experiences of children receiving hematopoietic stem cell transplant (HCT) and their caregivers, with a primary focus on the multifaceted aspects of adherence following discharge.

**Methods:**

Convenience sampling was used to enroll 14 caregivers and 15 children at a large Midwestern children’s hospital. Children had an allogenic HCT for a malignant or nonmalignant disorder and were 1–12 months off immunosuppression. Participants completed a semi-structured interview in the HCT-clinic or via phone about the child’s experience taking medications and adhering to post-transplant guidelines.

**Results:**

Caregivers were primarily female (*n* = 13, 87%), White (*n* = 11, 73%), and not Hispanic (*n* = 15, 100%). Children were primarily male (*n* = 9, 60%), White (*n* = 10, 67%; missing: *n* = 3), and not Hispanic (*n* = 13, 87%; missing: *n* = 2). Children’s average age was 13.14 years (*SD* = 2.88). Two primary themes emerged from the interviews, (1) *family navigation and self-management of post-HCT medications and restrictions* with 3 subthemes highlighting structured routines, adaptations to life post-HCT, and experiences with daily restrictions and other aspects of care; (2) *advice from families on navigating post-HCT care* with 2 subthemes highlighting communication and strategies for maneuvering post-HCT treatment. Of note, half of caregivers (*n* = 7, 50%) reported the child was responsible for taking their medications, and 43% (*n* = 6) of children were responsible for knowing when to take their medications.

**Significance of results:**

This study contributes a nuanced understanding of adherence in pediatric HCT, emphasizing the need for tailored interventions that transcend traditional medical frameworks and enable clear communication between families and the medical team. Findings underscore the importance of providers adopting a comprehensive and patient-centered approach. Healthcare providers should consider the psychosocial aspects of HCT, implement tailored family-centered strategies to optimize adherence, and prioritize comprehensive communication to improve outcomes.

## Introduction

Hematopoietic stem cell transplantation (HCT) is a transformative therapy for children experiencing malignant or nonmalignant disorders. While transplant offers hope for cure and/or improved quality of life, the success of HCT is tied to adherence (Pai et al. 2018) which, as defined by the World Health Organization, is “the extent to which a person’s behavior – taking medication, following a diet, and/or executing lifestyle changes, corresponds with agreed recommendations from a health care provider” (Sabaté 2003; Amonoo et al. 2023). Therefore, in the context of pediatric HCT, adherence extends beyond compliance with medication schedules. It encapsulates a commitment to infection control measures, dietary restrictions, and stringent follow-up regimens (Sabaté 2003). However, within pediatric HCT, current literature emphasizes *medication* adherence. For instance, adults report rates of medication nonadherence post-HCT from 0 to 75% (Khera et al. 2011; Morrison et al. 2017; Belaiche et al. 2021). In adolescents with cancer and post-HCT, rates range from 21 to 60% (Smith et al. 1979; Festa et al. 1992; Butow et al. 2010; McGrady et al. 2014; McGrady and Pai 2019). In pediatric HCT, rates range from 42 to 75% (Phipps and DeCuir-Whalley 1990; McGrady et al. 2014; Pai et al. 2018), increasing over time (McGrady et al. 2014; Morrison et al. 2017; Posluszny et al. 2022), and children miss at least 1 dose of medication 3 days a week (McGrady et al. 2014). Medication nonadherence has been associated with a higher incidence of infections in children post-HCT (Pai et al. 2018). While medication nonadherence poses clear risks, there is a gap in the literature regarding understanding the interplay of aspects of adherence beyond medication; therefore, this study aims to fill that gap.

Previous literature commonly examines barriers and facilitators in the context medication adherence; however, these barriers likely also affect other aspects of care. The most reported barrier is difficulty managing numerous medications (Hoegy et al. 2019; Chardon et al. 2022). Other barriers include long-term therapy, medication changes, transition of care (Hoegy et al. 2019), refusal, bad taste, administration method, and refills (Chardon et al. 2022). Medication barriers typically emerge after discharge and decrease over time (Chardon et al. 2022). Additionally, family caregivers are frequently unaware of potential post-HCT barriers before discharge (Chardon et al. 2022), making combatting barriers difficult. However, families may develop strategies to enhance their child’s adherence (facilitators), such as recognizing medication benefits, maintaining organization, social support, and establishing routines (Morrison et al. 2017; Hoegy et al. 2019). Age-related adherence facilitators include nasogastric (NG) tube for younger children and increased autonomy for adolescents (Chardon et al. 2022). Therefore, it is imperative to examine adherence barriers and facilitators, beyond medication, from both caregivers’ and children’s perspectives to help families post-HCT.

To broaden the understanding adherence post-HCT, examining both children and caregivers is essential because according to the Biobehavioral Family Model, biological, psychological, and social family factors impact individual and family health (Woods and Denton 2014). Furthermore, effective familial support and communication are integral for proper post-HCT care (Hoegy et al. 2019). For instance, a qualitative study in adults post-HCT found familial responsibilities as a barrier and caregiver support as a facilitator to adherence (Amonoo et al. 2023). Caregiver emotional well-being, treatment plan understanding, transition of care, and financial concerns affect pediatric adherence (Chardon et al. 2022). Family caregivers providing support, effective communication, trust, and high-quality bonds more effectively guide children post-HCT (Butow et al. 2010). Additionally, more social support for children decreases comorbidities and complications post-HCT (Nørskov et al. 2021). Simultaneously, the child’s age, cognitive development, and psychological resilience impact adherence (Butow et al. 2010). Therefore, various factors influencing the family caregiver–child relationship contribute to the child’s adherence, underscoring the need for a holistic understanding of adherence in pediatric HCT to inform tailored interventions addressing families’ needs.

This qualitative study explored the unique experiences of children and their caregivers, focusing on the multifaceted aspects of adherence post-HCT. While Amonoo et al. (2023) conducted a similar study in adults, it is important to recognize children face distinct challenges. Additionally, most adherence literature focuses on medication adherence. Therefore, a dyadic, qualitative approach was used to examine the psychosocial, familial, and healthcare factors influencing children’s adherence. The study aimed to (1) explore adherence barriers and facilitators for medications and restrictions/guidelines and (2) examine the experience of children and their caregivers with the complex post-HCT regimen. This study contributes to the existing HCT literature regarding barriers and facilitators and adds novelty by including the individual perspectives of children and qualitatively examining the multi-faceted aspects of HCT adherence.

## Methods

### Procedures

Eligible caregivers and children were recruited through the HCT outpatient clinic at a large Midwestern, free-standing children’s hospital (February–September 2020). During a routine clinic visit or via phone, a study team member obtained informed consent/assent and interviewed caregivers and children. This study was reviewed and approved by the local Institutional Review Board (#STUDY00000680).

### Participants

Twenty-nine participants were enrolled (14 caregivers and 15 children), representing 15 families. Most participants (*n* = 28) were matched caregiver–child dyads; however, the child sample includes 1 child who was 18 years old and their own caregiver. Children and caregivers meeting inclusion criteria, confirmed through a medical chart review, were approached during clinic visits. The inclusion criteria for children (8–18 years old) specified having an underlying malignant or nonmalignant disease, receiving an allogeneic HCT, and being at least 1–12 months off immunosuppression (patients typically wean off during the first 100 days post-transplant). Caregivers or children who were non-English speaking and/or had developmental delays were excluded. Convenience sampling was used to obtain a demographically diverse sample across age, sex, race/ethnicity, and diagnoses.

### Data collection

One trained nursing research assistant, 2 clinic nurses, and 1 doctorate-level nurse scientist conducted interviews using a semi-structured interview guide (Table 1). The interview guide was developed by 2 PhD-prepared investigators, the PI and psychologist (MS, CG), with a team of clinical experts. Through an iterative process, initial drafts were refined with expert feedback from HCT clinicians to ensure comprehensive content and clarity. Sample size was guided by the principle of data saturation and achieved when no new themes emerged, ensuring the depth necessary for comprehensive analysis (Hennink and Kaiser 2022).

**Table 1.** Pediatric HCT adherence interview guides

*Pediatric HCT adherence (caregiver) interview guide:* The purpose of this interview is to better understand your experience with care after transplant. We understand there are many medications, appointments, and guidelines for you to follow, and this can be difficult. We are going to ask you a few questions to get a better sense of your family’s experience during treatment. We will also describe an app for mobile phones that aims to help taking medications easier. We would like your feedback on the app and suggestions to make this type of program easier for families to use. Please know there are no right or wrong responses. We are interested in hearing your honest thoughts, so please share openly. We will be recording this discussion so we don’t miss any comments, but all of your answers will be kept confidential and anonymous.
First, tell me about your child’s experience taking medications during transplant? (How was it in the hospital and then after discharge?)
Tell me about your child’s experience taking immunosuppressant medication (like xyz) at home? (What challenges did you have taking immunosuppressants after discharge or what made it easier?)
Tell me about your experience with the guidelines/restrictions (like wearing a mask, avoiding school/crowds) after you were discharged home? (What challenges did you have following these guidelines after discharge or what made it easier?)
What advice do you have for other parents about administering medications and following restrictions/guidelines at home following transplant?
What would you like your healthcare provider to know about your experience with medications and restrictions/guidelines after discharge?
*Pediatric HCT adherence (child) interview guide:* The purpose of this interview is to better understand your experience with care after transplant. We understand there are many medications, appointments, and guidelines for you to follow, and this can be hard. We are going to ask you a few questions to get a better sense of what that is like for you and your family. We will also describe an app for cell phones that may make things easier. We would like your feedback on the app and ideas for us to make it better. Please know there are no right or wrong answers. We want to know what you think so that we can help other kids and families, please be honest. We will be recording this so we don’t miss any thoughts or ideas, but all of your answers will be kept confidential and anonymous.
First, tell me about your experience taking medications during transplant? (How was it in the hospital and then after discharge?)
Tell me about your experience taking immunosuppressant medication (like xyz) at home? (What challenges did you have taking immunosuppressants after discharge or what made it easier?)
Tell me about your experience with the guidelines/restrictions (like wearing a mask, avoiding school/crowds) after you were discharged home? (What challenges did you have following these guidelines after discharge or what made it easier?)
What advice do you have for other kids about taking medications and following restrictions/guidelines at home following transplant?
What would you like your doctors and nurses to know about your experience with medications and restrictions/guidelines after discharge?

### Data analysis

Demographic data were analyzed using descriptive statistics. Interview data were managed using NVivo 13 (2020, R1), a qualitative data analysis software, and analyzed using thematic analysis (Sundler et al. 2019). The analysis technique included (1) becoming familiar with the interviews, (2) generating initial codes, and (3) searching for themes. Child interviews were coded first, followed by caregiver interviews. The first 3 interviews from the child group and first 3 interviews from the caregiver group were analyzed using open coding. Two research team members (MS, KV) independently analyzed the remaining 23 interviews. Analysis was done by separating the dyads’ interviews by child and caregiver responses and analyzing them individually. Themes were derived across all child and caregiver interviews to provide the full picture of the family experience. However, few ideas were specific to the caregivers or children. After the themes were established, responses within dyads were associated and compared (Table 2) (Eisikovits and Koren 2010). Data analysis continued until thematic saturation was achieved. Saturation was monitored through discussions among the research team. To enhance credibility, member checking was conducted with a subset of caregivers (*n* = 5).

**Table 2.** Themes and exemplar quotes from caregivers and children dyads post-HCT

Exemplar quotations		
Theme	Dyad	
**Family navigation and self-management of post-HCT medications and restrictions**	**Caregiver**	**Child**
*Maintaining organization and a structured routine for medication management*	“It took a long time, it will take a long time to get through them, through so many” (mother of a 13-year-old boy).	“It’s just made real hard. There is a lot of pills and at different times of the day” (13-year-old boy).
	“We knew that if he took it and drank something real um … vibrant, like lemonade or coke or something that would kind of overwhelm the taste of it, he did better, as opposed to taking it with water” (mother of a 16-year-old boy).	“It was kind of easier to take them at the Ronald McDonald house since I could kind of space them out a little bit more” (16-year-old boy).
*Emotional, social, and practical adaptations to managing medications*	“He did very well, um because I was a nurse we were able to leave a little sooner and of course I was able to prepare him ahead of time about medications and make some suggestions as far as how to take certain medications that wouldn’t taste as bad and that sort of thing” (mother of a 16-year-old boy).	“I had a lot of medications to take so some of them were kind of hard to get them all down. And some would come back up. But, um, some of them weren’t really hard to take so, it wasn’t that bad” (16-year-old boy).
	“We had to do everything liquid. So, we have to crush whatever we cannot get liquid.”	“Yeah, I can’t swallow.”
	“It was horrific, was horrible, it was always screaming, chaotic. So, we had to come up with different ways to introduce the meds” (grandparent of a 9-year-old girl).	“I did not like it at all [taking medications]” (9-year-old girl).
*Managing aspects of care and daily restrictions that hinder post-HCT life*	“He felt very isolated and lonely and it was horrible and he couldn’t wait to go back to school and the school could not wait to get him back as well … you could tell he was going stir crazy (mother of 14-year-old boy).	“And being away from school, it does take a toll on you, just being away from everyone” (14-year-old boy).
	“I was able to talk him through, ya know, what it was going to be like and how he was gonna feel, ya know, when he lost his hair and those types of things … I had the background. I don’t know how people who don’t have that maneuver all of this” (mother of a 16-year-old boy).	“Sometimes wearing a mask in public is kind of just not really that fun to do because people just look at you. And I didn’t really want to go to school anyways, because if I had to wear a mask I just didn’t really want to. So, I was just home schooled that year” (16-year-old boy).
**Advice from families on navigating post-HCT care**		
*Communication as a cornerstone of care*	“The biggest advice I could give is definitely communicate with your team, you know, your BMT team. Let them know what’s going on with your child. Let them know your thoughts” (mother of a 15-year-old girl).	“I want to mention you want to talk about the worst-case scenario, even if it’s not common, but I still talk about that” (15-year-old girl).
	“Just talk to them, yeah, talk to your kids. That was the big thing. We were very open and we told her, you know, this is just how it is because of what you’re going through and all of that. So, she understood that. We made sure she was educated on that too” (mother of a 15-year-old boy).	“Definitely, to talk to them [medical team] and say what you think and not just have somebody else talk for you” (15-year-old boy).
*Practical strategies to maneuver post-HCT treatment*	“I think that is the best thing I could have done and accept help. A lot of people are like, no, I can do this myself. Well, it’s hard. It’s really hard” (mother of a 9-year-old girl).	“Well, one and done … I don’t have to do it again. If it makes them feel better, everybody has to take medicine” (9-year-old girl).
	“He was never on a PCA pump, he never had to have a feeding tube, we did all of the glucosamine and there’s a powder for his mouth sores. We started that in [home state] and I changed his toothpastes to limit his mouth sores. Ya know, we were proactive in hopes that we could avoid all of that and he did, he avoided all of it” (mother of a 16-year-old boy).	“I mean, they might not want to take it but you kind of really need to because in the end, it really helps. And it may seem like a lot, but it’s better than what you could be taking” (16-year-old boy).

## Results

Fourteen caregivers and 15 children (*n* = 29) participated. Demographic characteristics are described in Table 3 (caregivers) and Table 4 (children). The average length of caregiver interviews was 10 minutes (range: 5–18 minutes) and the average length of child interviews was 10 minutes (range: 3–18 minutes). One dyad completed the interview simultaneously (28 minutes). Additionally, half of the caregivers (*n* = 7, 50%) mentioned the child was responsible for managing their own medications and 43% (*n* = 6) indicated the child was responsible for knowing when to take them. Consistent across children and caregivers, the primary themes were (1) *family navigation and self-management of post-HCT medications and restrictions* and (2) *advice from families on navigating post-HCT care*. The first theme has 3 subthemes: (1a) *maintaining organization and a structured routine for medication management*, (1b) *emotional, social, and practical adaptations to managing medications*, and (1c) *managing aspects of care and daily restrictions that hinder post-HCT life*. The second theme has 2 subthemes: (2a) *communication as a cornerstone of care* and (2b) *practical strategies to maneuver post-HCT treatment*.

**Table 3.** Caregiver characteristics (*N* = 14)

	*N* (%)
**Gender**	
Male	1 (7%)
Female	13 (93%)
**Race**	
White	11 (78%)
Black or African American	2 (14%)
Asian	1 (7%)
**Parent’s ethnicity**	
Not Hispanic	14 (100%)
**Marital status**	
Single	5 (36%)
Married	9 (64%)
**Highest grade of school completed**	
High school	4 (29%)
College	8 (57%)
Graduate/Professional	1 (7%)
Missing	1 (7%)
**Annual family income**	
Under–$25,000 per year	1 (7%)
$25,001–$50,000 per year	4 (29%)
$50,001–$75,000 per year	3 (21%)
$75,001–$100,000 per year	1 (7%)
$150,001 or more per year	4 (29%)
Missing	1 (7%)
**Relationship to the child who is participating in this study**	
Biological parent	11 (78%)
Adoptive parent	1 (7%)
Grandparent	1 (7%)
Missing	1 (7%)

**Table 4.** Child characteristics (*N* = 15)

	*N*/Mean (%/SD)
**Child’s gender**	
Male	9 (60%)
Female	6 (40%)
**Child’s age (years)**	13.14 (2.88)
**Child’s race**	
White	10 (67%)
Black or African American	1 (7%)
Asian	1 (7%)
Missing	3 (20%)
**Child’s ethnicity**	
Not Hispanic	13 (87%)
Missing	2 (13%)
**Child’s time off immunosuppression (months)**	18.67 (9.76)
**Child’s educational status**	
Regularly attends school	8 (53%)
Is home-schooled	1 (7%)
Received homebound instruction or tutoring from the school	4 (27%)
Missing	1 (7%)

### Family navigation and self-management of post-HCT medications and restrictions

#### Maintaining organization and a structured routine for medication management

Caregivers and children highlighted the importance of a structured routine and staying organized to manage medications via (1) having a routine/chart, (2) following a schedule, (3) discovering a good method of administration, (4) using alarms/notifications, and (5) managing the quantity of medications. Eight caregivers and 2 children endorsed maintaining a routine and/or using a chart to stay organized, such as initially using a chart to create a routine, keeping the medications close to where they slept, putting out the week’s medications, or having a mental routine, “I had a routine in my head … her nurse gave us a chart, and she highlighted … she put morning, afternoon, evening … so that helped me early on because we went from being in the hospital where the nurses are around the clock and now mama got to do it” (mother of a 9-year-old girl). Caregivers (*n* = 4) expressed challenges following the medication schedule because of taking care of other children. Caregivers (*n* = 4) and children (*n* *=* 3) had difficulties remembering which medication to take when, “It’s just real hard. There is a lot of pills and at different times of the day” (13-year-old boy). Caregivers (*n* = 3) reported waking up their child as a barrier, “The actual pill taking wasn’t the issue. Waking him up was more an issue” (mother of a 15-year-old boy).

Another way to manage medications reported by caregivers (*n* = 4) and children (*n* = 4) was sticking with a successful method of administration. Caregivers reported using flavorful drinks, taking pills over liquid, or crushing up the pills into food or the NG tube made administration easier. Children reported taking medications at the Ronald McDonald house or in the hospital made it easier. Other children also reported using applesauce or a baby bottle to help, “I always make Mommy pretend like I was a baby and put it in like a baby bottle and I would take it like that. I don’t know why, but yeah, that’s just what was in my head” (9-year-old girl). Three caregivers and 1 child reported using phone notifications/alarms, “It became habit … but I always set an alarm just so I wouldn’t forget” (mother of a 15-year-old boy). Caregivers (*n* *=* 2) and children (*n* *=* 2) expressed difficulties keeping track of all the medications and how a successful management strategy helped.

#### Emotional, social, and practical adaptations to managing medications

While maintaining a routine and organization was a primary facilitator, caregivers and children described additional facilitators and barriers to medication adherence. They emphasized necessary adjustments for a successful transition to life post-HCT such as (1) combatting the bad taste of the medications, (2) managing side effects/symptoms, (3) acquiring knowledge of the process, (4) having parental help, (5) changing doses, (6) taking the medications at home, (7) setting goals, and (6) handling prescription refills. Caregivers (*n* = 5) and children (*n* = 1) reported the bad taste of medication as a barrier. Whether the child needed a flavorful drink, or the medication crushed into food, there was a shared sentiment that administering bad tasting medication was hard, “It was horrific, was horrible, it was always screaming, chaotic. So, we had to come up with different ways to introduce the meds” (grandparent of a 9-year-old girl). Another barrier reported by caregivers (*n* = 3) and children (*n* = 5) was medication symptoms/side-effects, such as lower extremity pain, vomiting, nausea, no appetite, thicker gums, hair growth, fevers, fatigue, and/or stomach pain, “Sometimes my body wouldn’t handle it well … sometimes I would throw it up. Sometimes I would be okay. Sometimes I just wouldn’t be able to take it, or it would take me a while to take it. So, it was kinda hard” (18-year-old male).

Three caregivers (no children) reported prior medical knowledge enabled them to handle the medications better, seek out necessary information, and “prepare [the child] ahead of time about medications and make some suggestions as far as how to take certain medications” (mother of a 16-year-old boy). Two children (no caregivers) discussed having their caregiver’s help made taking medications easier, “Having my mom there with me to help me measure it out” (14-year-old boy). One child (no caregivers) mentioned the difficulties of the doses changing and how “the doses were always awkward. Like they’d go sky high and then they had to go super low” (15-year-old boy). One child (no caregivers) also expressed how taking the medications at home and setting goals to take all the pills made it easier. One caregiver (no children) reported challenges keeping up with the prescription refills, “Trying to think, like, to get them refilled. I felt like I was always going to the pharmacy … that was the most challenging thing” (mother of a 9-year-old girl). Lastly, some caregivers and children reported easy adherence or no problems. Four children expressed that taking the medication wasn’t too bad, there was not a bad taste, and it was easy, “It wasn’t hard. I didn’t feel any different, and it didn’t taste too bad” (14-year-old boy); 6 caregivers reported facing no problems with their child taking their medications, “He didn’t really have any issues taking the medication” (mother of a 14-year-old boy).

#### Managing aspects of care and daily restrictions that hinder post-HCT life

While adherence to medication regimens poses challenges, the post-HCT guidelines and restrictions often introduce a variety of additional challenges. Most commonly, caregivers (*n* = 9) and children (*n* = 8) reported difficulties with the restricted social time at school and with friends. Across caregivers, there was a consensus that it “was a big challenge for [the child], not going back to school, not being able to get around [their] friends” (mother of a 15-year-old girl). Of the 8 children that reported limited social interactions, 1 expressed, “It wasn’t that big of a problem” (16-year-old boy), while 7 expressed it was hard, “I didn’t like that I wasn’t able to go to school, because I love seeing my friends and playing with them” (15-year-old boy). Additionally, 2 children (no caregivers) expressed their enjoyment of homeschool specifically, and how it benefitted them, “Being homeschooled was kind of an easy way to get my grades back into this year” (16-year-old boy). Two children and 2 caregivers also reported their child had difficulties feeling isolated from their inability to leave their house, “He felt very isolated and lonely and it was horrible, and he couldn’t wait to go back to school” (mother of a 14-year-old boy); “I felt lonely. I felt secluded, isolated” (14-year-old boy). In terms of wearing a mask, 1 caregiver and 5 children expressed difficulties and frustrations with wearing a mask and how having a routine helped, “You would have your mask with your baseball hat … if we’re going to go out, you grab your hat, and you’re in your mask” (mother of a 13-year-old boy); “I hate wearing a mask, but it’s something you just gotta do” (18-year-old male). One child (no caregivers) expressed that wearing a mask “is what it is, because you just have to wear the mask, and now everybody has to … I was just setting the trend” (11-year-old girl).

### Advice from families on navigating post-HCT care

#### Communication as a cornerstone of care

When asked about advice for future families and providers, caregivers and children reported various strategies centered around good communication: (1) involve your child, (2) communicate with the medical team, (3) maintain open communication, and (4) listen to doctors. Three caregivers (no children) expressed that it was helpful to involve their child in the treatment process; it “empowered [them] to take control of their own health” (mother of a 15-year-old girl), allowed children to understand their diagnosis and treatment. Three caregivers and 2 children described the importance of communicating with the medical team. Whether noting symptoms, sharing opinions, or leaning on them for support, caregivers shared the sentiment it was important “to keep good contact with the providers” (grandparent of a 9-year-old girl). The children suggested talking to the doctors so they know what is going on because, as 1 child shared, “if you don’t, you just end right back where you were at” (18-year-old male). One caregiver and 3 children advised families do as the doctors say, whether that be taking the medicine on time, keeping their hands clean, or staying away from sick people, “just basically follow the rules of the doctors” (14-year-old boy). Lastly, 3 caregivers and 1 child expressed the importance of the medical team fostering an open environment, “If you communicate in a certain way with your patient, they’ll be honest and then you can deal with it – just making them feel comfortable, to be honest with you” (mother of a 15-year-old girl).

#### Practical strategies to maneuver post-HCT treatment

When asked to offer advice to other families and providers, caregivers and children also shared practical strategies for handling life post-transplant: (1) remain hopeful, (2) be active, (3) stay busy indoors, (4) focus on other stuff, (5) make it a game, (6) rest, (7) trust, (8) find support, and (9) stay committed. Three children (no caregivers) expressed the importance of remaining hopeful, knowing normalcy would eventually return, and realizing “it will be over before you know it, and it could be way worse” (14-year-old boy). Two children and 1 caregiver advised finding ways to stay entertained indoors and be active, “don’t just sit there and make time just waste by” (15-year-old boy), even if that means you must wear a mask, “If I could have gone back, I would have worn a mask in public if I could’ve hung out with them. I would think they were judging me, but they really weren’t” (16-year-old boy). Additionally, 1 child (no caregivers) advised focusing on something else and making it a game, like “who can wear a mask and gloves longest” (9-year-old girl). Lastly, 1 child (no caregivers) expressed that rest is helpful, because “sometimes physical therapy and all the other stuff, really doesn’t help anymore and the only thing you want to do is rest” (14-year-old boy), and trust is important because “it makes me feel better when it is someone I trust” (9-year-old girl). Three caregivers (no children) advised families to find support and accept help, such as joining support groups on Facebook, relying on school resources, or welcoming help from family and friends. One caregiver (no children) advised sticking with the treatment even when it is challenging. When asked to give advice to future families, caregivers and children also provided suggestions that were previously addressed as common adherence facilitators: 6 caregivers (no children) said to maintain an organized routine, 2 children (no caregivers) said to discover a successful medication administration method, and 1 caregiver and 2 children said to manage the number/bad taste of medications and have assistance from the medical team regarding at-home medication administration.

## Discussion

This study uniquely examines pediatric HCT adherence, highlighting the multifaceted nature of adherence, beyond medication. The interviews revealed families found adherence success by having an organized medication system and finding practical ways to navigate life post-HCT. Families reported challenges with maintaining a structured routine, staying organized, and adapting to life post-HCT. Additionally, families shared valuable advice regarding the importance of communication and strategies to maneuver HCT treatment. Families also discussed the difficulties of post-HCT restrictions and interviews revealed children’s involvement. The child’s perspective also validates the need for social support from their medical staff, family, and friends post-HCT (Butow et al. 2010; Nørskov et al. 2021). This study provides concrete suggestions from children and their caregivers on navigating the restrictive HCT process, adhering to medical regimens, and making the most out of life post-HCT.

The first theme, *family navigation and self-management of post-HCT medications and restrictions*, addresses the first study aim by highlighting barriers, facilitators, and restrictions that families face post-HCT. The reported facilitators of medication adherence post-HCT were consistent with findings from previous studies. For instance, maintaining routines and understanding the benefits of the medications were reported facilitators (Hoegy et al. 2019). Additionally, families shared specific adherence strategies, including using an alarm system, medication chart, and unique methods of medication administration. Consistent with previous literature, the most frequently reported barriers included the number of medications (Hoegy et al. 2019; Chardon et al. 2022; Amonoo et al. 2023), medication distaste, method of medication administration (Chardon et al.), complex medication schedule, and medication side effects (Amonoo et al. 2023). Beyond the expected medical and pharmacological aspects, psychosocial, familial, and healthcare system factors play a significant role in adherence challenges. The dynamic interplay of these factors is emphasized through the nuanced narratives from the children and their caregivers and highlight a need for holistic, individualized approaches.

The second theme, *advice from families on navigating post-HCT care*, addresses the second aim by highlighting collaborative advice and communication between the child, medical team, and caregiver. While few differences emerged between caregiver and child reports within themes, the individual perspectives from the children present unique information about their communication preferences, feelings about transplant, and strategies for managing life post-HCT. Additionally, individual findings from caregivers reveal the treatment responsibility between caregivers and their child. Half of caregivers reported the child was responsible for making sure they took their medications and just under half of the children were responsible for knowing when to take the medication. However, it is notable that the level of responsibility and involvement the child had with their medications (i.e., was there supervision?) remains unclear. In literature, caregivers report difficulties managing medication responsibility with their partners and assuming full responsibility for the medication regimen post-HCT (Chardon et al.). Additionally, adolescents with positive family relationships, open communication, and strong social support have better adherence and fewer complications (Nørskov et al. 2021), whereas poor relationships have been associated with adherence challenges (Butow et al. 2010). Therefore, literature has begun to shed light on the unique child-caregiver dynamics related to medication responsibility post-HCT and associated barriers and/or facilitators; this study illuminates that reality as the results include shared medication responsibility and emphasizes communication. The involvement of the child in medication responsibilities underscores the importance of communication throughout the transplant process and when considering future interventions.

### Limitations

While this study provides valuable insights, certain limitations merit acknowledgment. The single-center nature of the study and the potential for recall bias are important considerations. The diversity in caregivers was limited as they were primarily female, White, and well-educated, and child diversity was limited by race. The interview guides targeted medication management and post-HCT restrictions/guidelines, not family dynamics, a common topic that emerged. Additionally, the study was conducted during the COVID-19 pandemic, which could have made the families’ experiences handling the treatment regimens and restrictions (i.e., avoiding crowds, wearing masks) easier than other times. Therefore, prospective mixed-method studies are needed to confirm findings. Despite the aforementioned limitations, the sample had diverse representation of socioeconomic statuses, included non-parental caregivers, met saturation, and ample information was provided to evaluate study aims. Future research should explore the generalizability of these findings across healthcare settings, delve into the implications of shared child-caregiver medication responsibility, and consider the multifaceted aspects of adherence. Additionally, education, employment, marital, sibling, and socioeconomic status of families in relation to their adherence should be examined.

### Conclusion and clinical implications

Findings underscore the importance of adopting a comprehensive and patient-centered approach in pediatric HCT care due to the complexity of navigating treatment and life post-HCT. All individuals involved should be attuned to the child’s needs and support system. Healthcare providers should know the family’s psychosocial structure, incorporate family-centered strategies, and enhance communication. For instance, providers can facilitate regular family meetings to explore family concerns, clarify roles, and set realistic self-management goals. Interdisciplinary team members can play specific roles in supporting families: nurses can provide education and assess family readiness; social workers can address emotional barriers for effective self-management; dietitians can involve families in culturally-tailored nutrition planning. These practices can promote family engagement to improve HCT outcomes.
